# Use of modern contraceptives among married women in Vietnam: a multilevel analysis using the Multiple Indicator Cluster Survey (2011) and the Vietnam Population and Housing Census (2009)

**DOI:** 10.3402/gha.v9.29574

**Published:** 2016-02-29

**Authors:** Lan Thi Hoang Vu, Juhwan Oh, Quyen Thi-Tu Bui, Anh Thi-Kim Le

**Affiliations:** 1 Department of Epidemiology and Biostatistics, The Hanoi School of Public Health, Hanoi, Vietnam; 2 JW LEE Center for Global Medicine, Seoul National University College of Medicine, Seoul, Korea

**Keywords:** contraception, family planning, reproductive health, maternal health, differentials, multilevel modeling, MDG

## Abstract

**Background:**

The prevalence of modern contraceptive use is an important indicator that reflects accessibility to reproductive health services. Satisfying unmet needs for family planning alone could reduce the number of maternal deaths by almost a third. This study uses multiple data sources to examine multilevel factors associated with the use of modern contraceptives among married women in Vietnam aged 15–49 years.

**Design:**

Data from different national surveys (Vietnam Population and Housing Census, Vietnam Living Standard Survey, and Multiple Indicator Cluster Survey) were linked to create a dataset including individual and contextual (provincial) variables (*N=*8,341). Multilevel modeling was undertaken to examine the impact of both individual and provincial characteristics on modern contraceptive use. Odds ratios (ORs) and 95% confidence intervals (CIs) are reported.

**Results:**

Individual factors significantly associated with the use of modern contraceptives were age 30–34 years (reference 15–19 years) (OR=1.63); high socioeconomic status (SES) (OR=0.8); having two living children (OR=2.4); and having a son (OR=1.4). The provincial poverty rate mediated the association between the individual's SES and the likelihood of using modern contraceptives.

**Conclusions:**

The proportion of women in Vietnam using modern contraceptive methods has remained relatively high in recent years with significant variation across Vietnam's 63 provinces. Women of lower SES are more likely to use modern contraceptive methods, especially in the poorer provinces. Achieving access to universal reproductive health is one of the Millennium Development Goals. Vietnam must continue to make progress in this area.

## Introduction

The use of modern contraceptive methods is an important indicator that reflects accessibility to reproductive health services (1). The contraceptive prevalence rate among women aged 15–49 years is indicator 5.3 in the 5th Millennium Development Goal (MDG) (2). Singh and Darroch (3) asserted that increasing the use of modern contraceptive methods is crucial for achieving three of the MDG targets – improving maternal health, reducing child mortality, and combating HIV/AIDS. Overall increased use of contraceptives and improved reproductive health can contribute to achieving all eight MDGs (3).

Vietnam has made significant achievements in family planning programs in recent decades (4). The country still has a relatively young population with the proportion of women of reproductive age (15–49 years) at about 29%. Yet in recent years, international aid for family planning programs in Vietnam has been reduced (4). Developing and implementing ways of sustaining and improving the widespread use of contraceptives in Vietnam is a challenge for policy makers.

Recent research shows that more than one-third of unmarried women have unmet needs for contraception, especially for modern methods. Factors such as educational level and socioeconomic status (SES) are associated with the use of modern contraceptives (5–9). It is also suggested that contextual factors, such as the SES of the community in which a woman resides, may affect their contraceptive use (10, 11).

Although factors affecting contraceptive use in Vietnam have been investigated in past studies, to our knowledge, no previous studies have used nationally representative samples to examine the independent impact of community factors on contraceptive use. This study uses nationally representative data from the Vietnam Multiple Indicator Cluster Survey (MICS), the Vietnam Population and Housing Census (VPHC), and the Vietnam Living Standard Survey (VLSS) to examine the independent impact of contextual and individual factors on the use of modern contraceptives by Vietnamese women of reproductive age. Contextual effects are investigated at the provincial level, as has been done in previous studies (12).

## Methodology

### Data sources

Data analyzed in this article came from two sources:

1. The Vietnam MICS: This survey was conducted in 2010–2011 by the General Statistics Office of Vietnam aligning with the fourth global round of MICS (MICS 4). MICS 2011 comprises information collected from a sample of 11,614 households and provides a comprehensive picture of children and women in Vietnam's six regions. For the purposes of this article, we extracted contraceptive information from the dataset of married women aged 15–49 years living in the selected households (*N*=8,341) (13). The term ‘married’ includes all women in a conjugal union.
2. The VPHC 2009 and the VLSS 2008: These datasets provided the provincial data for the multilevel analysis (14, 15).

Records from each data source were linked using ‘province’ as the key identifier.

### Measurements

#### Study outcome

Modern contraceptive use was measured according to whether the sampled women reported whether they, or their partners, were using a ‘modern contraceptive method’. These methods included sterilization (female or male), intra-uterine devices, implants, injectable contraception, male or female condoms, and contraceptive pills. This variable was binary whereby one denoted ‘use of any modern contraceptive methods’ and zero denoted ‘do not use any modern contraceptive methods’.

#### Level 1 independent variables

Individual variables selected for inclusion in level 1 of the model included current place of residence (urban vs. rural), ethnic group (Kinh group vs. other ethnic groups), women's highest completed education level (no formal school vs. primary school vs. lower secondary vs. upper secondary vs. college/university), number of living children (less than two children vs. two children vs. three or more children), having a living son or not, women's age group in years (15–19, 20–24, 25–29, 30–34, 35–39, 40–44, or 45–49), and SES (low, middle, or high).

The variable used to denote SES was derived from a wealth index score using principal component analysis. This index was created by using information related to the household's wealth such as the ownership of consumer goods, dwelling characteristics, water, and sanitation. Factor weights (factor scores) were assigned to household assets, and each household was given a wealth index score. The scores were categorized into tertiles, representing low, middle, and high household wealth or SES.

#### Province-specific characteristics (level 2)

Information on provincial characteristics was extracted from the VPHC (2009) and the VLSS (2008) (13, 14). Province-specific characteristics were analyzed under the domains ‘economic environment’, ‘fertility stabilization’, and ‘educational status’, which are frequently reported in the literature as ‘macro’ level determinants of population fertility or contraceptive use. No new data were collected.

The economic environment domain was measured using two variables:

*Provincial poverty rate:* This is measured as the percentage of poor households in the province according to Vietnamese standards.

*Income inequality:* ‘Income inequality’ is measured as the ratio between the average monthly income per capita of the highest quintile group (quintile 5) and the lowest quintile group (quintile 1); the bigger the ratio, the higher the income inequality within the province.

The fertility stabilization domain included two indicators:

*Total fertility rate (TFR):* TFR of a province is the number of children, on average, born to a woman over her lifetime. The measure is obtained by summing single-year age-specific birth rates at a given time.

*Percentage of women aged 15*–*49 who had a third child:* This is the percentage of women aged 15–49 years, who had a third or subsequent child during the 12 months prior to the survey, among all women aged 15–49 who gave birth during that period. Because Vietnam has maintained the ‘two children policy’ in the last few decades, in the last 7 years there has been a strong commitment to ‘regulate’ married couples who may want to have three or more children. Three or more children per woman equates to an above-replacement fertility rate.

The educational status domain included two indicators:

*Percentage of the population who attained a minimum of college or university level education:* This measure was the percentage of population who reported that their highest completed education was college or higher among the population aged 15 and over.

*Percentage of the population with no formal schooling:* This refers to the percentage of the population who reported that they had never attended school among the population aged 5 and over.

### Statistical method

This study used multilevel analysis. Multilevel modeling can 1) examine the independent effects of individual and contextual variables on the likelihood of using modern contraceptives, 2) control for the effects of clustering of subject-specific measures within provinces, and 3) evaluate cross-level interaction between individual and provincial level factors (16, 17). The data source included survey weights. It has been suggested that when undertaking multilevel modeling, analysts should scale design weights and fit models using unweighted and scaled-weighted data (18, 19). We used hierarchical linear modeling to analyze both scaled-weighted data and unweighted data. However, we found that weighted and unweighted data did not diverge significantly in general (i.e. the estimated coefficients and standard errors of all variables were not different between weighted and unweighted models).

The study analyzed a dichotomous outcome (contraceptive use). The multilevel model uses a binomial sampling model and a logit link. The following model building strategy was used to build a multilevel model to estimate the likelihood of married women, aged 15–49 years, in Vietnam, using modern contraceptive methods.

- *Step 1:* The ‘empty’ or ‘null model’ with no explanatory variables was used to determine whether there was an overall difference among provinces in terms of the likelihood of using modern contraceptives (i.e. whether the intercept was random, or in other words, if the variance component of the intercept was significant).
- *Step 2:* The ‘individual’ model included level 1 independent variables to allow assessment of the association between the individual characteristics and modern contraceptive use. Level 1 variables were entered one at a time with a random slope. If a significant variance component of the slope was reported, the variable was retained with a random slope. Otherwise, the variable was constrained as fixed across the provinces (i.e. the level 1 variable was considered to have a fixed slope if variance component of the slope was not significant).
- *Step 3:* The third or ‘final’ model, which included explanatory variables at both levels (level 1 and level 2), was fitted to test for the net effect of characteristics at province level, as well as the cross-level interaction between level 1 and level 2 variables (i.e. the slope of the level 1 variable was modeled as a function of the level 2 variable).

## Results

### Trends in contraceptive use over three rounds of MICS

**Fig. 1:**
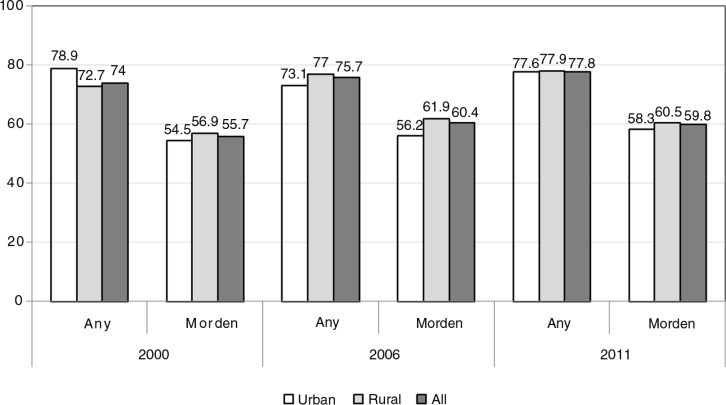
Trends in contraceptive use across three rounds of MICS (2000, 2006 and 2011). Individual variables selected for inclusion in level 1 of the model included: current residence (urban vs. rural); ethnic group (Kinh group vs. other ethnic groups); women's education level (no school, primary school, lower secondary, upper secondary, and college/universities); number of living children (less than two; two children; three or more); having a living son; women's age (age group 15–19, 20–24, 25–29, 30–34, 35–39, 40–44, and 45–49 years); and SES (low, middle, and high).

Figure 1 shows the trend in contraception use. In general, the trends remained unchanged. The proportions of women using modern contraceptive methods in 2000, 2006, and 2011 were 55.7, 60.4, and 59.8%, respectively. In all three rounds, the proportions using modern contraceptives among women living in rural areas were slightly higher than those for women in urban areas.

### Characteristics of women aged 15–49 in MICS sample in 2011

Table 1 presents the characteristics of the respondents. About 10% were aged 20–24 years, and approximately one-third were aged over 40 years. In terms of residence, 29.2% of respondents resided in urban areas and most (87.2%) were of Kinh ethnicity. About 31% had upper secondary school education (including college/university), and only 4.7% of had no schooling. The proportion of women with a living son was 71.6%. About 70% had two or more children and 6.4% had no children. In term of contraception, about 78% of married women aged 15–49 used some form of contraception. Of these, 59.8% used modern methods and 17.9% used traditional methods.

**Table 1:** Descriptive analysis of individual variables

Variable		*n*	%
Ethnicity: Kinh		7,274	87.2
Residence: living in urban		2,436	29.2
	15–19	143	1.7
	20–24	828	9.9
Age	25–29	1,498	18.0
	30–34	1,643	19.7
	35–39	1,530	18.3
	40–44	1,456	17.5
	45–49	1,244	14.9
	No school	396	4.7
	Primary school	1,626	19.5
Education	Lower secondary school	3,739	44.8
	Upper secondary school	1,413	16.9
	College/university	1,167	14.1
Having any living son		5,975	71.6
	No child	537	6.5
Number of living children	1 child	1,977	23.7
	2 children	3,883	46.5
	3 children or more	1,944	23.3
	Any modern method	4,992	59.8
Use of contraceptive	Any traditional method	1,495	17.9
	Any method	6,487	77.8

### Characteristics of provinces in Vietnam

The sample size at level 2 was 63 provinces. Table 2 presents the descriptive characteristics of the 63 provinces in Vietnam. The average provincial poverty rate was 18.8% (SD=11.4). The percentage of women having a third child within the past year in each province ranged from 7.2 to 39.2%.

**Table 2:** Descriptive analysis of provincial variables

Variables	*n*	Minimum	Maximum	Mean	Standard deviation
Percentage of population aged five and over with no formal schooling	63	2.0	33.2	7.1	7.2
Percentage of population who had attained a minimum of college level education	63	1.1	13.2	3.1	2.2
Percentage of women aged 15–49 who had a third child	63	7.2	39.2	17.1	8.2
Total fertility rate	63	1.45	3.45	2.16	0.38
Income inequality (Q5/Q1)	63	5.06	9.18	6.81	0.93
Provincial poverty rate	63	0.5	58.2	18.8	11.4

### Determinants of modern contraceptive use in the MICS sample in 2011

The multilevel model showed that both individual and provincial factors had impacts on the likelihood of modern contraceptive use among married women in Vietnam, aged 15–49 years. Of the seven individual variables included in the analysis, four showed a significant association with the outcome, these being women's age, SES, number of living children, and having a son; and three variables (ethnicity, residence, and education status) did not have any association with the outcome. Table 3 presents the final model that assesses the likelihood of using modern contraceptives.

**Table 3:** Final multilevel model for use of modern contraceptives among married women aged 15–49 years in Vietnam

Variable	Coefficient (standard error)	Odds ratio (95% CI)	*p*
Age 15–19	–	–	–
Age 20–24 versus 15–19	1.16 (0.28)	3.2 (1.8–5.5)	<0.01
Age 25–29 versus 15–19	1.50 (0.27)	4.5 (2.6–7.5)	<0.01
Age 30–34 versus 15–19	1.63 (0.28)	5.1 (2.9–8.9)	<0.01
Age 35–39 versus 15–19	1.38 (0.28)	4.0 (2.3–7.0)	<0.01
Age 40–44 versus 15–19	1.15 (0.28)	3.2 (1.8–5.4)	<0.01
Age 45–49 versus 15–19	0.56 (0.27)	1.7 (1.0–2.9)	0.05
Have no son	–	–	–
Any living son	0.36 (0.07)	1.4 (1.2–1.6)	<0.01
Have less than two children	–	–	–
Have 2 children	0.88 (0.06)	2.4 (2.1–2.7)	<0.01
Have ≥3 children	0.83 (0.09)	2.3 (1.9–2.7)	<0.01
Low SES	–	–	–
Middle SES	−0.12 (0.07)	0.9 (0.7–1.1)	0.07
High SES	−0.21 (0.08)	0.8 (0.7–0.9)	<0.01
Living in urban	–	–	–
Living in rural	−0.07 (0.06)	0.92 (0.8–1.1)	0.18
Women's education level: no school	–	–	–
Primary school	0.07 (0.12)	1.1 (0.9–1.4)	0.59
Lower secondary school	0.10 (0.12)	1.1 (0.9–1.4)	0.40
Upper secondary	0.16 (0.13)	1.2 (0.9–1.5)	0.23
College/universities	0.06 (0.13)	1.1 (0.8–1.4)	0.63
Kinh	–	–	–
Other ethnic groups	0.12 (0.08)	1.1 (0.94–1.3)	0.2
Interaction between high SES and provincial poverty rate	−0.013 (0.006)		0.04
Random intercept	0.22 (*χ* ^2^=129.8, df=62, *p<*0.001)		
Random slope of variable high SES	0.03 (*χ* ^2^=95.5, df=661, *p*<0.001)		

In general, the relation between women's age and the use of modern contraceptives was an inverted U shape, in which the likelihood of modern contraceptive use was low among women aged 15–24, higher among women aged 25–34, and lower for women aged 35 years and above. Having more children, or having a son, was significantly associated with a higher likelihood of using modern contraceptives. There were no differences in the likelihood of using modern contraceptives between women in the low and middle SES tertiles (*p*=0.07). However, compared to women in the low SES tertile, women in the high SES tertile had a lower likelihood of using modern contraceptives (*p<*0.01).

The variance component for the intercept was 0.22 (*χ* ^2^=129.8, df=62, *p<*0.001) indicating that the random part of the intercept was highly significant. The variance component for the slope of the high SES group was 0.03 (*χ* ^2^=95.5, df=61, *p<*0.001), and the slope of the high SES variable was significant, which means that the association between high SES and modern contraceptive use varied across the 63 provinces. The final multilevel model showing the likelihood of using modern contraceptives was a random intercept/slope model.

**Fig. 2:**
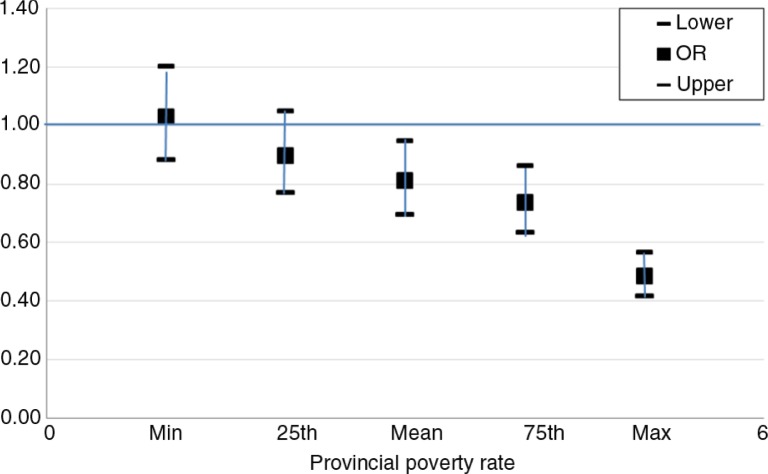
Odd ratios for contraceptive use in high SES compared to low SES according to the provincial poverty rate.

A cross-level interaction between high SES and the provincial poverty rate was identified (*p*=0.04). To assist the interpretation of this interaction, we estimated the odds ratio (OR) of the high SES corresponding to the provincial poverty rate in Fig. 2.

Figure 2 provides the ORs of using modern contraceptive methods among women with high SES compared to those with low SES for different values of the provincial poverty rate (min, 25th percentile, mean, 75th percentile, and max). It was estimated that for women living in a better-off province (i.e. with a low rate of poverty), those with high SES did not have lower likelihood of using modern contraceptives compared to women with low SES. However, as the provincial poverty rate increased, the differences between these two groups became significant.

## Discussion

The definition of contraceptive use is consistent across three MICS rounds in 2000, 2006, and 2011 and similar to that in MDG 5 (13, 20, 21). This provided an opportunity to assess changes over time in the use of modern contraceptive methods. These results show that the proportion of pregnant women or those having sexual partners’ using modern contraceptive methods still remains high and has changed little between 2000, 2006, and 2011. This finding is possibly a result of the Vietnam strategy for population and reproductive health remaining fairly stable over this time period.

The results showed that the likelihood of married women aged 15–49 years using modern contraceptive methods varied significantly across Vietnam's 63 provinces. This study found significant association between women's age and numbers of living children. Specifically, contraceptive use had an inverted U shape relationship with mother's age (whereby women aged 25–35 had the highest likelihood of contraceptive use) and the more children a woman had, the higher the likelihood of contraceptive use. These findings are similar to previous studies in Vietnam and other countries (22–25).

The results also indicate that the status of having a living son affected women's use of contraceptive methods. According to several studies, this was also reflected through social preferences for having a son in the family (26). This reflects culture and tradition and also sexual inequality (27). The UNFPA reports that sex inequality disfavoring women can affect their decisions about contraceptive use at the household level (28).

It was interesting to find that women of lower SES had a higher likelihood of using modern contraceptives. Some previous studies also confirmed this association and indicated that this may be explained by the national family planning program in countries that target mostly poor women (29). This may be considered a success of targeted government-sponsored family planning programs aimed at socioeconomic equality in contraceptive use in Vietnam. In addition, this is also consistent with research in other middle-income countries showing that the ‘higher provision of public capital may compensate for low levels of human capital, i.e. SES, in regard to modern contraceptive use’ (30).

More significantly, provincial SES (i.e. the poverty rate) moderated the association between household SES and the likelihood of modern contraceptive use. The reverse association became stronger in poorer provinces (i.e. provinces with a higher rate of poverty). This can also be explained by the fact that, in Vietnam, many sponsored family planning programs have focused more strongly on low-income households in selected disadvantaged provinces. Previous studies have reported contextual effects to material differences between geographical areas (e.g. environmental quality and health services) or to negative psychological impacts (the prevalence of prevailing attitudes toward health and health-related behaviors) (31, 32). Association between geographical SES and the likelihood of using contraceptives has also been reported elsewhere (10, 11, 29). However, to our knowledge, this is the first study to report the mediating effect of provincial SES on the association between individual SES and the likelihood of using contraceptives. More broadly, this finding adds to the growing evidence that contextual factors can have independent effects on the health of a local population.

### Strengths and limitations

The analyses were undertaken on the most recently available MICS dataset (2011) therefore providing reasonably current estimates. The definitions and indicators used here are consistent and comparable with those used both in Vietnam and other countries. The multilevel methods allowed us to assess factors at both community and individual levels in order to gain insights into factors associated with contraceptive use.

However, our study has some shortcomings. First, the MICS data are cross-sectional meaning that it is not possible to infer cause and effect. Second, the use of secondary data did not allow us to analyze other factors (e.g. cultural or social) not included in the dataset that may have had some association with contraceptive use. This is reflected in the significant variance component of the intercept in the final model, after taking all contextual/individual variables into account. The surveyed women might or might not have used contraceptive methods for other reasons not included in this analysis. Therefore, the findings need to be explained and interpreted with caution. In addition, our study linked surveys conducted at different time points and this may have created discrepancies. The contextual data (VPHC and VHSS) were collected prior to MICS data.

## Conclusions

To summarize, the use of modern contraceptive methods in Vietnam has remained relatively high with some change between 2000 and 2011. The likelihood of married women using modern contraceptives varies significantly across Vietnam's 63 provinces. Achieving access to universal reproductive health is one of the Millennium Development Goals. Vietnam must continue to make progress in this area, which can also help prevent maternal and newborn deaths (33).
